# Association of TNF-Alpha, MBL2, NOS2, and G6PD with Malaria Outcomes in People in Southern Ghana

**DOI:** 10.1155/2022/6686406

**Published:** 2022-02-28

**Authors:** Godfred Futagbi, Paulina S Otu, Mubarak Abdul-Rahman, Ebenezer K Aidoo, Aminata C Lo, Ben A Gyan, Yaw A Afrane, Linda E Amoah

**Affiliations:** ^1^Department of Animal Biology and Conservation Science, College of Basic and Applied Sciences, University of Ghana, Accra, Ghana; ^2^Department of Medical Microbiology, University of Ghana Medical School, University of Ghana, Accra, Ghana; ^3^Department of Pathology, University of Ghana Medical School, University of Ghana, Accra, Ghana; ^4^Department of Medical Laboratory, Accra Technical University, Accra, Ghana; ^5^Immunology Department, Noguchi Memorial Institute for Medical Research, University of Ghana, Accra, Ghana; ^6^Department of Medical Parasitology, Faculty of Medicine, University Cheikh Anta Diop, Dakar, Senegal; ^7^West Africa Center for Cell Biology of Infectious Pathogens, University of Ghana, Accra, Ghana

## Abstract

**Background:**

One major issue that has set back the gains of the numerous malaria control interventions that national malaria control programs have implemented is asymptomatic malaria. Certain host genetic factors are known to influence symptomatic malaria; however, not much is known about how host genetics influences the acquisition of asymptomatic malaria.

**Methods:**

Genomic DNA was extracted from whole blood collected from 60 symptomatic and 149 nonfebrile (asymptomatic, *N* = 109, and uninfected, *N* = 40) volunteers aged between 2 and 69 years from a high (Obom) and a low (Asutsuare) malaria transmission setting in Southern Ghana. Restriction fragment length polymorphism (RFLP) was used to determine polymorphisms at the MBL2 54, TNF-*α* 308, NOS2 954, and G6PD 202/376 gene loci.

**Results:**

Polymorphisms at the MBL2 54 and TNF-*α* 308 loci were significantly different amongst the three categories of volunteers in both Asutsuare (*p* = 0.006) and Obom (*p*=0.05). In Asutsuare, a low malaria transmission area, the allele *G* has significantly higher odds (3.15) of supporting asymptomatic malaria as against symptomatic malaria. There were significantly higher odds of TNF-*α* genotype GA being associated with symptomatic malaria as against asymptomatic malaria in both sites, Obom (*p*=0.027) and Asutsuare (*p*=0.027). The allele B of the G6PD gene was more prevalent in symptomatic rather than asymptomatic parasite-infected individuals in both Obom (*p*=0.001) and Asutsuare (*p*=0.003).

**Conclusion:**

Individuals in Southern Ghana carrying the TNF-*α* 308 GA genotype are more likely to exhibit symptoms of malaria when infected with the malaria parasite as opposed to harboring an asymptomatic infection. Also, the B allele of the G6PD gene is likely to prevent a *P. falciparum*-infected person from exhibiting symptoms and thereby promote asymptomatic parasite carriage.

## 1. Background

Millions of individuals, particularly children and expectant mothers, suffer from malaria in areas of Africa known for *Plasmodium falciparum* endemicity. Ghana is one of the countries hardest hit by malaria, accounting for about 2% of global malaria cases. Despite successes in malaria control in Ghana over the past decade, there were 161 cases per 1000 of the population at risk as of 2019 [1]. Infections of humans with *Plasmodium falciparum (P. falciparum)* can result in different manifestations of malaria: asymptomatic and symptomatic. Symptomatic cases could be uncomplicated or complicated, and complicated cases could be severe malaria or cerebral malaria [2]. Asymptomatic malaria infections are highly frequent in malaria-endemic areas, specifically in high and intermediate transmission settings [3], where they remain undiagnosed and untreated [4]. The use of molecular assays such as polymerase chain reaction (PCR) to detect parasite DNA has enhanced the diagnosis of asymptomatic infections [5], which often present at submicroscopic densities.

Persistent exposure to *Plasmodium* parasites in high transmission areas results in premunition (partial immunity) and the subsequent ability to maintain asymptomatic parasite carriage within a given population [4]. A very large number of *P. falciparum* infections that are detected during community surveys are asymptomatic [6]. These asymptomatic infections have the potential to enhance malaria transmission by constantly producing gametocytes, which are not effectively cleared by the components of the artemisinin combination therapy (ACT) used as a first-line treatment of malaria in sub-Saharan African countries, including Ghana [7]. It is thus very important to identify all possible causes and contributors including host genetics to the asymptomatic carriage of *P. falciparum* parasites in order to reduce the transmission and incidence of malaria.

In symptomatic malaria, the effects of polymorphisms in some genes, including the beta globulin gene (HBB), glucose 6 phosphate dehydrogenase gene (G6PD), mannose-binding lectin gene (MBL2), tumor necrotic factor-alpha (TNF-*α*) gene, and inducible nitric oxide synthase 2 (NOS2), on providing protection from or enhancing susceptibility to the disease have been identified [8–15]. Not much, however, is known about the contributions of host genetics to asymptomatic malaria [16]. A large population-based study using methods similar to those used to identify the genetic determinants of severe malaria would be an ideal tool that could identify the genetic determinant of asymptomatic malaria [15]. However, in the absence of such large studies, a probe into the possible influence of some selected genes that have been suggested to alter susceptibility to severe malaria can provide insight into the genetic determinants of asymptomatic malaria. G6PD is an essential enzyme in the pentose phosphate pathway. Polymorphisms in the G6PD gene, including A376 G and G220 A, can result in reduced enzyme activity that leads to a condition referred to as G6PD deficiency [17, 18]. Deficiency in the G6PD gene is suggested to be protective against malaria [19]; however, a recent screen identified polymorphisms in the G6PD gene to be associated with a significant risk of severe malaria [15]. One large-scale multicentre genetic study identified increasing levels of G6PD deficiency to be associated with decreasing risk of cerebral malaria but increasing in risk of severe malaria anaemia [20]. Polymorphisms in the G6PD gene are in linkage disequilibrium with the 202 mutations, and all were found to be associated with severe malaria [20].

Mannose-binding lectin gene (MBL2) is a soluble pathogen-recognizing molecule that is involved in the activation of the complement system of the innate defence mechanisms. Point mutations in the promoter region and exon 1 of the MBL2 result in mannose-binding lectin (MBL) deficiency. Single nucleotide polymorphisms (SNPs) in MBL2, including G54 A, are known to result in reduced levels of functional MBL2 protein in circulation [21], which causes MBL deficiency. Fluctuations in the serum concentration of MBL result from different genotypes of MBL2, with heterozygotes having 10% functional activity and the homozygote mutant exhibiting less than 1% functional activity [22]. Mannose-binding lectin recognizes molecules such as mannose and N-acetyl-glucosamine on different microorganisms, including *P. falciparum*, and upon binding activates the complement system through the interaction with MBL-associated serine proteases (MASP-1, -2, and -3 and Map 19) and kills the potential pathogen by the membrane attack complex and complement-mediated phagocytosis [23] or assists in phagocytosis through the release of proinflammatory cytokines [24, 25]. However, contradictory reports have been observed between MBL2 deficiency and malaria [16, 21].

Tumor necrotic factor-alpha (TNF-*α*) is an inflammatory cytokine that results in apoptosis and necrosis and is also a mediator of tumor regression, an effector of cachexia amongst other functions [26]. SNPs in the regulatory region of the TNF gene are associated with TNF production as well as the clinical outcomes of malaria in diverse populations [27]. Polymorphism at position 308 in the promoter region of the TNF-*α* gene is known to result in increased production of TNF-*α* [18], with the 308A allele (TNF2) known to activate the production of TNF more than the 308G allele (TNF1) [28, 29]. Studies in Gabon found out that TNF −308G > *A* polymorphism has less space for recurrent malaria [30]. Other studies have also shown an association of TNF-308 GA heterozygous (TNF1/2) state with severe *falciparum* malaria [31]. TNF-*α* has been related to antiparasitic activity, and persistently high levels of this cytokine lead to a rapid reduction in parasitaemia [32].

Inducible nitric oxide synthase 2 (NOS2) gene produces nitric oxide (NO), and polymorphisms in the promoter region, including a point mutation at position 954, have been suggested to exhibit enhanced NOS2 activity [12]. There are conflicting reports of the influence of NOS2 gene polymorphisms on malaria severity, where some studies reported the absence of an influence [31, 33] and another identified a decreased risk of severe malaria [34]. The NOS2 promoter variants are suggested to be candidates for the clinical malaria outcome [35, 36]. Differences observed in the role polymorphisms of selected genes play in resistance or susceptibility to malaria could be influenced by immunity and ethnicity [37]. This study sought to determine whether polymorphisms in selected regions of the G6PD, MBL2, TNF-*α*, and NOS2 genes influence the manifestation of *P. falciparum* parasite carriage in adults and children living in Southern Ghana. These polymorphisms were selected because they were found to be associated with the severity of malaria, and perhaps, they might play a role in asymptomatic parasite carriage. This study will serve as a basis for larger studies that probe into identifying the role host genetics plays, especially in the manifestation of asymptomatic malaria, and contribute to revising policies on the management of asymptomatic infection in endemic countries, including Ghana.

## 2. Methods

### 2.1. Study Site

The study was conducted in two communities in the Greater Accra Region of Southern Ghana, Asutsuare and Obom [38]. Obom is located in the Ga South District, where malaria transmission is perennial but peaks during the major rainy season between June to August [39]. Asutsuare is located in the Shai-Osudoku District (125 km from Obom), with low but seasonal malaria transmission, which increases slightly after the rainy season between April to July [40, 41].

### 2.2. Study Design and Sampling

The samples for this study included 150 archived samples collected from afebrile (who did not exhibit any signs and symptoms of malaria) individuals as part of a cross-sectional community survey conducted in January/February 2016 (during the off-peak season) in both Obom (*N* = 84) and Asutsuare (*N* = 66). Another 60 study participants with microscopy-confirmed *P. falciparum* parasites and malaria-related symptoms, including a fever of ≥37.5 C at the time of sample collection, were recruited between March and September 2016 from the Obom Health Center (*N* = 40) and the Osodoku Community Health Center (*N* = 20) in Asutsuare.

Venous blood (1 ml) was collected from each participant into EDTA tubes. A drop (5 *µ*l) of blood was used to spot the Urit 12 (Accurex Biomedical Private Limited, India) hemoglobin meter according to the manufacturer's instructions.

### 2.3. Extraction and Quantification of DNA from Whole Blood

Extraction of DNA was carried out on whole blood using the Quick-gDNA Mini Prep extraction kit (Zymo Research, USA) using the recommended manufacturer's protocol. Briefly, 400 *µ*l of genomic lysis buffer was added to 100 *µ*l of blood, vortexed, and allowed to settle at room temperature for 5–10 minutes. The solution was then transferred into a Zymo-Spin Column in a collection tube and centrifuged for a minute. The columns were then washed with DNA prewash buffer (200 *µ*l) followed by a wash buffer (500 *µ*l). Afterwards, 90 *µ*l of elution buffer was added to the column to elute the DNA. The eluted genomic DNA (gDNA) was subsequently quantified using a nanodrop 2000C and either used immediately or stored at −20°C.

### 2.4. *Plasmodium falciparum* Species Identification

*Plasmodium falciparum* species identification was done using nested PCR [42] as previously described by Amoah et al. [43, 44] with minor modifications. The primary amplification reaction contained the genus-specific primers, rPlus 5 and rPlus 6 (Additional File Table S1) at a final concentration of 10 *µ*M with 30–50 ng of gDNA. The secondary nested amplification utilized *P. falciparum* species-specific primers (Fal1 and Fal2, Additional File Table S1) also at 10 *µ*M per reaction, each with 0.5 *µ*l of the primary reaction product. The total volume for both reactions was 15 *µ*l supplemented with 0.5 U of Taq Polymerase (NEB, UK). MRA102 g, gDNA extracted from the 3D7 *P. falciparum* strain was used as a positive control and distilled water (dH_2_O) as the negative control. The secondary PCR products were electrophoresed on 1.5% agarose gel containing 0.5 *µ*g/ml ethidium bromide and subsequently visualized under UV, using a gel imager (Vilber, Germany).

### 2.5. Gene Polymorphism Analysis

Restriction fragment length polymorphism (RFLP) was used to determine all the gene polymorphisms, using previously published procedures [16, 44] with minor modifications. Briefly, the regions of interest in the MBL2, TNF-*α*, G6PD, and NOS2 genes were amplified in a 25 *µ*l PCR reaction mixture containing 15–30 ng of genomic DNA, 200 nM of each primer (MBL21 and MBL22, TNF1 and TNF2, and 376F and 376R; 202F and 202R and NOS21 and NOS22) (Additional File Table S1), and 0.5 U of OneTaq Polymerase (NEB, UK). The amplification was performed using the Mastercycler nexus (Eppendorf, USA) and annealing temperatures set at 61.6°C, 58°C, 60°C, 65°C, and 60°C, respectively. A list of all primers and restriction enzymes used is in Additional File Table S1.

All PCR amplicons were digested for 1 hr using 5 U of the appropriate restriction enzyme at the recommended temperature. The digested products were electrophoresed as described above, with the exception that the agarose gel was set at 2% (Additional File S2 Figure 1).

### 2.6. Statistical Analysis

Data analysis was carried out using SPSS version 22.0. Percentage and frequency distribution were calculated for gender, age, and parasite prevalence estimated by both microscopy and PCR. The Chi-square test or Fisher's exact test was used to determine significant differences amongst the different categories for each of the genes. Statistical significance was defined as *p* < 0.05.

## 3. Results

### 3.1. Demographic Data of the Study Population

The 209 study participants were aged between 2 and 69 years in Asutsuare and 6 to 60 years in Obom, and there was no significant difference between the mean age of participants from the two sites (*p*=0.118). A total of 150 participants comprising 69% (58/84) and 77% (51/66) of the afebrile individuals from Obom and Asutsuare, respectively, tested positive for malaria parasites by microscopy and/or PCR and were classified as asymptomatic (AS). The remaining 31% (26/84) and 23% (15/66) of the afebrile individuals from Obom and Asutsuare, respectively, were free of malaria parasites and classified as uninfected (UN). The mean (SEM) age of the participants across the three malaria groups in both sites ranged between 20.9 (1.9) and 25.8 (2.4) years, with no statistical difference observed (*p*=0.960 and 0.092 in Obom and Asutsuare, resp.; Table 1). The distribution of female participants between the sites did not vary significantly (*p*=0.390). The male to female ratio was also similar between the two sites (*p*=0.889). The prevalence of female participants ranged from 47% to 65% and was not significantly different amongst the three groups (*p*=0.311) (Table 1); however, the ratio of males to females (M/F) varied significantly amongst the three groups (*p* < 0.05), and this was due to marked difference between the uninfected and the asymptomatic groups. The distribution of the participants amongst the three groups in both sites did not vary significantly (*χ*^2^ = 1.06, *p*=0.593, for Obom and *χ*^2^ = 2.07, *p*=0.357, for Asutsuare). Hemoglobin data were available only for the asymptomatic and the uninfected group; the levels ranged between 12 g/dl and 13 g/dl and were uniformly distributed between the groups (*p*=0.533 and 0.292 in Obom and Asutsuare resp.; Table 1). The mean temperature was similar between the asymptomatic and the uninfected groups in both sites (*p*=0.701 and 0.152 in Obom and Asutsuare, resp.). All the participants with symptomatic malaria had a fever of 37.5°C or higher (Table 1).

### 3.2. Mannose-Binding Lectin (MBL2) 54 Genotype and Malaria Status

In Obom, the wild-type MBL 54 AA allele was the most prevalent variant in all the three (asymptomatic, AS; symptomatic, S, and uninfected, UN) groups, and no significant difference (*p*=0.534) was observed in the distribution of allelic variants across the three groups (Table 2). The wild-type allele was the most prevalent amongst the asymptomatic and uninfected groups in Asutsuare, whilst significant differences in the distribution of the allelic variants amongst the three groups were observed (*p*=0.006). In Asutsuare, the MBL2 54 GA genotype had significantly higher odds of supporting asymptomatic infections compared to no infection (*p*=0.021) (Table 3). The odds of developing symptomatic malaria in Asutsuare were also found to be lower in individuals with the *G* allele compared to asymptomatic parasite carriage (*p*=0.036). In other words, allele *G* has significantly higher odds (3.15) of supporting asymptomatic malaria compared to symptomatic malaria in individuals from Asutsuare. In Obom, however, there was no significant difference in the odds of any of the MBL2 54 mutant genotypes or alleles supporting asymptomatic or symptomatic malaria (Table 3).

## 4. Tumor Necrotic Factor-Alpha (TNF-*α*) 308 Genotype and Malaria Status

In Obom, marginal differences were observed in the distribution of the TNF-*α* 308 variants amongst the three malaria groups (*p*=0.05) (Table 2). In Asutsuare, there was no significant difference in the distribution of the TNF-*α* 308 variants across the various malaria groups (*p*=0.178). There were significantly higher odds of TNF-*α* 308 genotype GA supporting symptomatic malaria compared to asymptomatic malaria in both sites, Obom (*p*=0.027) and Asutsuare (*p*=0.027) (Table 3). Additionally, in Obom, allele A had significantly lower odds of supporting asymptomatic malaria relative to no infection (*p*=0.034) (Table 3).

### 4.1. Nitrogen Oxide Synthase 2 (NOS2) 954 Genotype and Malaria Status

In Obom, there was, however, no significant difference in the distribution of the NOS 954 variants amongst the three malaria groups (Table 2). Similarly, in Asutsuare, no significant difference was observed in the distribution of the variants amongst the three malaria groups (*p*=0.342). Also, in both Asutsuare and Obom, there were no significant differences in the odds of the three NOS2 954 genotypes supporting asymptomatic or symptomatic malaria compared to no infection (Table 3).

### 4.2. Glucose 6 Phosphate Dehydrogenase (G6PD) 202 and 376 Genotypes and Malaria Status

The G6PD genotypes identified in this study were grouped into two: the deficient “d” group, with the presence of one or more A- genotypes and included A-A-, AA-, BA-, and A-, and the normal “n” group, with no A- genotype and included A, AA, B, BB, and AB. This resulted in the AS group comprising 8d and 101n participants, the S group comprising 7d and 53n participants, and the UN group comprising 1d and 40n (1d and 24n from Obom and 16n from Asutsuare) participants as distributed in Table 2. No significant difference was observed in the distribution of the variants in Obom (*p*=0.541); however, a significant difference was observed in Asutsuare (*p*=0.042), most likely due to the very low prevalence of G6PD deficiency amongst the people in Asutsuare. In both Asutsuare and Obom, there was no significant difference in the odds of the G6PD “d” group supporting asymptomatic or symptomatic infections relative to no infection (Table 4). However, there were significantly higher odds of the allele B supporting asymptomatic infection compared to symptomatic malaria in both sites, 2.89 times in Obom and 4.17 times in Asutsuare.

## 5. Discussion

Asymptomatic carriage of *P. falciparum* is a major obstacle to malaria control [45], and the identification of individuals who are highly prone to carry parasites in asymptomatic infections is of utmost importance. In countries with limited resources available for malaria control, identifying host genotypes that are associated with asymptomatic parasite carriage within the population can help in selecting the target population for certain control interventions. The influence of host genetics on malaria has been well studied over the past years; however, these studies mainly determine the influence on symptomatic malaria [46], with very few studies identifying how host genetics, particularly certain host gene polymorphisms, can influence asymptomatic *P. falciparum* parasite carriage [47]. This study was set out to determine whether some host factors that are known to protect people living in malaria-endemic settings from symptomatic malaria predispose them to harbor *P. falciparum* in asymptomatic infections. As such, polymorphisms in G6PD, MBL2, TNF-*α*, and NOS2 genes in a group of *P. falciparum*-infected and *P. falciparum*-uninfected adults and children living in a low and a high malaria transmission setting in Ghana were characterized.

Significant variations in the distribution of MBL2 54 and G6PD variants were observed amongst the volunteers sampled from only the low transmission setting, Asutsuare. The variations observed in the distribution of G6PD variants are most likely due to the very low prevalence of G6PD deficiency amongst the people in Asutsuare, which could suggest that the prevalence of G6PD deficient (G6PDd) variants decreased with decreasing transmission intensity. Studies have shown a higher frequency of G6PDd variants in high endemic areas explaining the fact that G6PDd variants increased with increasing transmission intensity, whereas the prevalence of G6PD deficiency in the high transmission setting, Obom, was higher and did not vary significantly amongst the different groups of people sampled in this study.

The data show that MBL2 54 GA genotype and allele *G* support asymptomatic parasite carriage in Asutsuare, which is a low malaria transmission area, where the MBL 54 GA genotype was associated with a 4-fold increase in the likelihood of a person harboring malaria parasites in an asymptomatic infection compared to the individual not having an infection. The mutant *G* allele also increased the likelihood of individuals having an asymptomatic infection by 3-fold relative to exhibiting symptoms of malaria when infected with the malaria parasite. The association of the mutant allele with asymptomatic infection is not surprising because the mutant exhibits much lower functional activity compared to the wild type, and considering the role played by MBL2 in complement activation and innate defence mechanisms [20], it is expected that parasite clearance would be defective as a result. None of these associations were observed in the high transmission setting, suggesting that transmission intensity has a significant role to play in the acquisition and maintenance of the asymptomatic infection. However, reports from studies conducted on children from a high transmission setting in Gabon [11] and in India [48] show associations of polymorphisms at MBL2 54 with exhibiting symptoms of severe malaria. This suggests that the outcome of MBL2 54 mutations and MBL deficiency is influenced by the intensity of *P. falciparum* transmission. Some studies have identified the TNF-*α* 308AA to be associated with severe malaria [49], whilst others found no association of TNF-*α* 308 mutations with malaria parasite carriage [50]. In a study from Gabon, no association was identified between TNF-*α* 308 GA and asymptomatic malaria amongst nonfebrile children [16], and another report from Nigeria also indicated that the TNF-*α* 308 GA was not able to differentiate between asymptomatic and symptomatic malaria [30]. In this study, a significant association was identified between symptomatic malaria and the TNF-*α* 308 GA mutation. TNF-*α* 308 GA resulted in a significant increase in the odds of a person infected with malaria parasites exhibiting symptoms (being symptomatic) in both low and high malaria transmission settings. This suggests that the TNF-*α* 308 GA mutation makes a person susceptible to symptomatic malaria episodes irrespective of the level of malaria transmission in the community. TNF-*α* 308 GA mutation increases the production of TNF [21, 22], and reports indicate that overproduction of TNF leads to severe malaria [51–53], hence the manifestation of symptoms. The advantage of this is that individuals with TNF-*α* 308 GA would immediately seek treatment for malaria as they would exhibit symptoms and not add to the transmission reservoir of asymptomatic carriers. This will subsequently result in a reduction of the infectious reservoirs associated with asymptomatic parasite carriage. Additionally, in the high malaria transmission area (Obom), the mutant allele A of TNF-*α* 308 was found to significantly reduce the risk of asymptomatic infection compared to no infection in afebrile individuals. This also supports a reduction in the prevalence of transmission reservoirs.

The G6PD deficiency status of the volunteers in this study was not associated with malaria outcomes, symptomatic, asymptomatic, or uninfected. One likely reason for this outcome is that the G6PD genotype does not always correlate with the phenotype (enzyme activity), such that some people with a genetic deficiency possess normal enzyme activity [54, 55]. This phenomenon has led to a number of studies conducted in malaria-endemic countries, including Ghana [44] and Uganda [56], reporting results similar to those found in this study. However, there were significantly higher odds of the wild-type allele B supporting asymptomatic infection compared to symptomatic malaria in both sites. The allele B produces normal enzyme activity. Functional G6PD enzyme activity is known to protect cells from oxidative damage through activities of antioxidants such as glutathione [57], which might protect infected red blood cells as well as the parasite, but it is not clear how this might promote asymptomatic malaria.

Polymorphisms at the NOS2 954 loci were similarly distributed in the three categories of volunteers and were not associated with disease outcomes. No reports exist on the association of NOS2 954 genotype with asymptomatic or symptomatic malaria; however, some findings point to its association with disease severity, though they are inconsistent [12, 58]. The dynamics of the acquisition of antidisease and antiparasite immunity to malaria are important confounding factors that influence malaria parasite carriage and exhibition of malaria symptoms during the course of an infection. A larger longitudinal study involving active and passive monitoring of *P. falciparum* carriage, episodes of symptomatic malaria, and the development of immunity in the study volunteers is required to validate the observations made in this study.

## 6. Conclusion

*Plasmodium falciparum*-infected individuals living in both high and low malaria transmission settings who carry the TNF-*α* 308 GA genotype are more likely to exhibit symptoms of malaria, whilst those with the B allele of the G6PD gene are likely to remain asymptomatic. Significant differences in the distribution of MBL 54 variants were observed amongst volunteers from the low transmission area, where the MBL 54 GA genotype was found to be associated with a 4-fold increase in asymptomatic malaria parasite carriage amongst afebrile volunteers.

Put together, our data suggest that host genetics may play a role in asymptomatic malaria parasite carriage and, in effect, the sustenance of malaria transmission. Thus, migration and the general movement of individuals into and out of especially low transmission settings can significantly influence the dynamics and patterns of malaria transmission.

### 6.1. Limitations

Due to logistical constraints, the monitoring of *P. falciparum* carriage by asymptomatic volunteers was not done beyond five days. This means that we could not rule out the possibility of some of the asymptomatic volunteers developing symptoms thereafter. The uneven distribution of females in the groups could have caused a bias in the distribution of G6PD genotypes identified in this study.

## Figures and Tables

**Table 1:** Demographic characteristics of the study population.

Site	Asutsuare (*n* = 84)	Obom (*n* = 125)		*P* value
Age (yrs) (mean (SEM))	25.18 (2.1)	21.97 (1.19)		0.118
Gender (F) (*n* (%))	40 (46.5)	65 (52.4)		0.390
M/F ratio	1.10	0.908		0.889
Malaria status	Uninfected	Asymptomatic	Symptomatic	*P* value
Overall (*n* (%))	41 (19.5)	1 (51.9)	60 (28.6)	<0.001
Gender (F) (*n* (%))	26 (65.0)	51 (46.8)	35 (58.3)	0.311
M/F ratio	0.538	1.137	0.714	<0.05
Age (yrs)				
Mean (SEM)	25.8 (2.4)	23.6 (1.4)	20.9 (1.9)	0.2550
Min–max	8 to 57	3 to 60	2 to 69	
Hb (g/dl)				
Mean (SEM)	12.5 (0.7)	12.2 (0.4)	ND	0.602 a
Min–max	4.0 to 20.3	4.8 to 20.2		
Temp/^o^C (mean (SEM))	36.7 (0.1)	36.5 (0.1)	≥37.5	0.059a
Obom, *n* (%)	26 (20.0)	58 (46.8)	40 (32.2)	0.593
Age (years)				
Mean (SEM)	22.0 (2.6)	22.2 (1.8)	21.5 (2.1)	0.960
Min–max	9 to 55	6 to 60	7 to 45	
Hb (g/dl)				
Mean (SEM)	12.0 (0.4)	12.3 (0.3)	ND	0.533 a
Min–max	7.2 to 16.2	8.7 to 20.0		
Temp/^o^C (mean (SEM))	36.7 (0.1)	36.6 (0.1)	>37.5	0.701 a
Asutsuare (*n* (%))	15 (17.4)	51 (59.3)	20 (23.3)	0.357
Age (years)				
Mean (SEM)	32.3 (4.2)	25.1 (2.1)	19.8 (3.9)	0.092
Min–max	9 to 57	3 to 59	2 to 69	
Hb (g/dl)				
Mean (SEM)	13.0 (1.0)	12.1 (0.4)	ND	0.292 a
Min–max	4.0 to 20.3)	4.8 to 20.2		
Temp/^o^C (mean (SEM))	36.7 (0.1)	36.3(0.3)	>37.5	0.152 a

M, male; F, female; SEM, standard error of the mean; n, the total number of samples; min, minimum value; max, maximum value; ND, not determined; a, comparisons made between asymptomatic and the uninfected groups only.

**Table 2:** Distribution of genotypes.

Site	Gene	Genotype	Uninfected	Asymptomatic	Symptomatic	*χ*2	*P* value
Obom	*MBL2 54*	GG	6	11	8	3.17	0.534
		GA	3	9	10		
		AA	16	39	20		
	*TNF 308*	AA	19	7	4	9.48	0.05
		GA	20	14	15		
		GG	18	18	5		
	*NOS 954*	CC	7	8	5	3.4	0.491
		GC	24	12	12		
		GG	25	14	7		
	*G6PD*	A	7	11	8	11.03	0.683
		A-	0	3	3		
		A-A-	0	0	0		
		AA	2	6	8		
		AA-	1	3	1		
		B	6	15	6		
		BA	6	9	4		
		BA-	0	1	0		
		BB	3	11	10		
		d	7	4	1	1.25	0.541
		n	52	36	24		

Asutsuare	*MBL2 54*	GG	3	12	3	14.55	0.006
		GA	0	11	10		
		AA	13	27	6		
	*TNF 308*	AA	13	3	3	6.3	0.178
		GA	14	3	9		
		GG	21	9	4		
	*NOS 954*	CC	14	5	3	4.31	0.342
		GC	8	5	7		
		GG	21	8	5		
	*G6PD*	A	4	13	5	20.32	0.124
		A-	0	1	0		
		A-A-	0	0	1		
		AA	1	4	6		
		AA-	0	0	2		
		B	5	15	3		
		BA	3	8	2		
		BA-	0	0	0		
		BB	3	9	1		
		d	0	1	3	6.4	0.042
		n	16	49	17		

AS, asymptomatic; S, symptomatic; UN, uninfected. Variants for the genes: AA = wild type, GA = heterozygous, and GG = homozygous for MBL2 54; GG = wild type, GA = heterozygous, and AA = homozygous for TNF 308; GG = wild type, GC = heterozygous mutant, and CC = homozygous for NOS 954; d, G6PD deficient genotype group; *n*, G6PD normal genotype group; G6PD genotypes: A-, deficient (d) male; A or B, normal (*n*) male; A-/A-, homozygous deficient (d) female; A/A- or B/A-, heterozygous deficient (d) female; A/A, B/B, or B/A, normal (*n*) female.

**Table 3:** Association between MBL2 54, NOS2 954, or TNF-*α* 308 genotype and malaria status.

Gene	Genotype/allele		Uninfected (UN)	Asymptomatic (AS)		Symptomatic (S)	UN versus AS		UN versus S		AS versus S	
	N (%)						OR (95% CI)	*P* value	OR (95% CI)	*P* value	OR (95% CI)	*P* value
MBL2 54Obom	AA	39 (66.1)			20 (52.6)	16 (64.0)	Reference		Reference		Reference	
	GA	9 (15.3)			10 (26.3)	3 (12.0)	2.17 (0.76–6.19)	0.144	0.81 (0.19–3.40)	1	0.38 (0.09–1.60)	0.175
	G	31 (0.26)			26 (0.34)	15 (0.30)	1.46 (0.78–2.73)	0.237	1.20 (0.58–2.50)	0.617	1.21 (0.56–2.62)	0.624
	A	87 (0.74)			50 (0.66)	35 (0.70)						

Asutsuare	AA	27 (54.0)			6 (31.6)	13 (81.2)	Reference		Reference		Reference	
	GA	11 (22.0)			10 (52.6)	0	4.09 (1.19–14.01)	0.021				
	G	37 (0.37)			16 (0.42)	6 (0.19)	1.24 (0.58–2.65)	0.584	0.39 (0.15–1.04)	0.055	0.32 (0.11–0.95)	0.036
	A	63 (0.63)			22 (0.58)	26 (0.81)						

TNF-*α* 308 Obom	GG	18 (31.6)			18 (46.2)	5 (20.8)	Reference		Reference		Reference	
	GA	20 (35.1)			14 (35.9)	15 (62.5)	0.70 (0.27–1.80)	0.458	2.70 (0.82–8.93)	0.098	3.58 (1.13–13.19)	0.027
	A	73 (0.51)			28 (0.36)	23 (0.48)	0.54 (0.31–0.96)	0.034	0.89 (0.47–1.72)	0.741	1.64 (0.79–3.41)	0.182
	G	71 (0.49)			50 (0.64)	25 (0.52)						

Asutsuare	GG	21 (43.7)			9 (60.0)	4 (25.0)	Reference		Reference		Reference	
	GA	14 (29.2)			3 (20.0)	9 (56.2)	0.50 (0.11–2.18)	0.492	3.38 (0.87–13.13)	0.071	6.75 (1.16–39.20)	0.027
	A	40 (0.42)			9 (0.3)	15 (0.47)	0.60 (0.25–1.45)	0.252	1.24 (0.55–2.76)	0.603	2.06 (0.72–5.85)	0.173
	G	56 (0.58)			21 (0.7)	17 (0.53)						

NOS2 954	GG	25 (44.6)			14 (41.2)	7 (29.2)	Reference		Reference		Reference	
	GC	24 (42.9)			12 (35.3)	12 (50.0)	0.89 (0.34–2.32)	0.823	1.79 (0.60–5.30)	0.294	2.00 (0.60–6.71)	0.259

Obom	C	38 (0.34)			28 (0.41)	22 (0.46)	1.36 (0.73–2.54)	0.327	1.65 (0.83–3.28)	0.154	1.21 (0.57–2.55)	0.617
	G	74 (0.66)			40 (0.59)	26 (0.54)						
	GG	21 (48.8)			8 (44.4)	5 (33.3)	Reference		Reference		Reference	

Asutsuare	GC	8 (18.6)			5 (27.8)	7 (46.7)	1.64 (0.41–6.54)	0.719	3.68 (0.90–15.01)	0.083	2.24 (0.45–11.11)	0.320
	C	36 (0.42)			15 (0.42)	13 (0.43)	0.99 (0.45–2.18)	1	1.06 (0.46–2.46)	0.888	1.07 (0.40–2.85)	0.888
	G	50 (0.58)			21 (0.58)	17 (0.57)						

AS, asymptomatic; S, symptomatic; UN, uninfected. Variants for the genes: AA = wild type, GA = heterozygous, and GG = homozygous for MBL2 54; GG = wild type, GA = heterozygous, and AA = homozygous for TNF 308; GG = wild type, GC = heterozygous mutant, and CC = homozygous for NOS 954.

**Table 4:** Association between glucose 6 phosphate dehydrogenase (G6PD) 202 and 376 genotypes and malaria status.

G6PD	Genotype	Uninfected (UN)	Asymptomatic (AS)	Symptomatic (S)	UN versus AS	*P* value	UN versus S	*P* value	AS versus S	*P* value
	N (%)				OR (95% CI)					
Obom	d	1 (4.0)	7 (11.9)	4 (10.0)	3.23 (0.38–27.75)	0.426	2.67 (0.28–25.34)	0.641	0.857 (0.234–3.14)	1
	n	24 (96.0)	52 (88.1)	36 (90.0)						
	Total	25	59	40						
	Allele									
	B	18 (0.49)	47 (0.53)	30 (0.48)	1.39 (0.63–3.33)	0.420	0.47 (0.21–1.03)	0.056	0.35 (0.19–0.65)	0.001
	A-	1 (0.02)	7 (0.08)	4 (0.06)	3.70 (0.42–32.52)	0.262	1.14 (0.12–11.11)	1	0.31 (0.08–1.12)	0.099
	A	18 (0.49)	34 (0.39)	63 (0.46)						

Asutsuare	d	0	1 (2.0)	3 (15.0)					8.65 (0.84–88.82)	0.067
	n	16 (100.0)	49 (98.0)	17 (85.0)						
	Total	16	50	20						
	Allele									
	B	14 (0.61)	41 (0.58)	7 (0.22)	0.91 (0.35–2.38)	0.842	0.21 (0.06–0.71)	0.010	0.24 (0.09–0.63)	0.003
	A-	0	1 (0.01)	4 (0.12)					5.52 (0.58–53.05)	0.165
	A	9 (0.39)	29 (0.41)	21 (0.66)						

d, G6PD deficient genotype group (containing at least one deficient A allele); n, G6PD normal genotype group (not containing any deficient A alleles).

## Data Availability

All data generated or analyzed during this study are included in this published paper and its supplementary information files.

## Abbreviations

MBL: Mannose-binding lectin
TNF: Tumor necrosis factor
NOS2: Nitric oxide synthase
G6PD: Glucose 6 phosphate dehydrogenase
G6PDd: Glucose 6 phosphate dehydrogenase deficient
AS: Asymptomatic
S: Symptomatic
UN: Uninfected.

## Supplementary Materials

Supplementary Materials Additional File S1 Table 1. Primers and restriction enzymes used for *P. falciparum* species identification and genotyping. Additional File S2 Figure 1. Representative agarose gel images of selected PCR reactions.

## References

[1] WHO (2020). *World Malaria Report 2020: 20 Years of Global Progress and Challenges*.

[2] Chen I., Clarke S. E., Gosling R. (2016). Asymptomatic” malaria: a chronic and debilitating infection that should Be treated. *PLOS Medicine*.

[3] Crookston B. T., Alder S. C., Boakye I. (2010). Exploring the relationship between chronic undernutrition and asymptomatic malaria in Ghanaian children. *Malaria Journal*.

[4] Lindblade K. A., Steinhardt L., Samuels A., Kachur S. P., Slutsker L. (2013). The silent threat: asymptomatic parasitemia and malaria transmission. *Expert Review of Anti-infective Therapy*.

[5] Alves F. P., Gil L. H. S., Marrelli M. T., Ribolla P. E. M., Camargo E. P., Da Silva L. H. P. (2005). Asymptomatic carriers of Plasmodium spp. as infection source for malaria vector mosquitoes in the Brazilian Amazon. *Journal of Medical Entomology*.

[6] Bousema T., Okell L., Felger I., Drakeley C. (2014). Asymptomatic malaria infections: detectability, transmissibility and public health relevance. *Nature Reviews Microbiology*.

[7] Karl S., Laman M., Moore B. R. (2015). Gametocyte clearance kinetics determined by quantitative magnetic fractionation in melanesian children with uncomplicated malaria treated with artemisinin combination therapy. *Antimicrobial Agents and Chemotherapy*.

[8] Roberts D. J., Williams T. N. (2003). Haemoglobinopathies and resistance to malaria. *Redox Report*.

[9] López C., Saravia C., Gomez A., Hoebeke J., Patarroyo M. A. (2010). Mechanisms of genetically-based resistance to malaria. *Gene*.

[10] Driss A., Hibbert J. M., Wilson N. O., Iqbal S. A., Adamkiewicz T. V., Stiles J. K. (2011). Genetic polymorphisms linked to susceptibility to malaria. *Malaria Journal*.

[11] Luty A. J. F., Kun J. F. J., Kremsner P. G. (1998). Mannose-binding lectin plasma levels and gene polymorphisms in Plasmodium falciparumMalaria. *The Journal of Infectious Diseases*.

[12] Kun J. F., Mordmüller B., Perkins D. J. (2001). Nitric oxide synthase 2lambaréné (G-954C), increased nitric oxide production, and protection against malaria. *The Journal of Infectious Diseases*.

[13] McGuire W., Hill A. V. S., Allsopp C. E. M., Greenwood B. M., Kwiatkowski D. (1994). Variation in the TNF-*α* promoter region associated with susceptibility to cerebral malaria. *Nature*.

[14] Opi D. H., Swann O., Macharia A., Uyoga S., Band G., Ndila C. M. (2018). Two complement receptor one alleles have opposing associations with cerebral malaria and interact with alpha (+) thalassaemia. *Elife*.

[15] Ndila C. M., Uyoga S., Macharia A. W. (2018). Human candidate gene polymorphisms and risk of severe malaria in children in Kilifi, Kenya: a case-control association study. *The Lancet. Haematology*.

[16] Mombo L.-E., Krishnamoorthy R., Bisseye C. (2003). Human genetic polymorphisms and asymptomatic Plasmodium falciparum malaria in Gabonese schoolchildren. *The American Journal of Tropical Medicine and Hygiene*.

[17] D’Alfonso S., Richiardi P. M. (1994). A polymorphic variation in a putative regulation box of the TNFA promoter region. *Immunogenetics*.

[18] Ahmad T., Wallace G. R., James T. (2003). Mapping the HLA association in Behçet’s disease: a role for tumor necrosis factor polymorphisms?. *Arthritis & Rheumatism*.

[19] Ruwende C., Khoo S. C., Snow R. W. (1995). Natural selection of hemi- and heterozygotes for G6PD deficiency in Africa by resistance to severe malaria. *Nature*.

[20] Clarke G. M., Rockett K., Kivinen K. (2017). Characterisation of the opposing effects of G6PD deficiency on cerebral malaria and severe malarial anaemia. *Elife*.

[21] Garred P., Larsen F., Seyfarth J., Fujita R., Madsen H. O. (2006). Mannose-binding lectin and its genetic variants. *Genes and Immunity*.

[22] Hibberd M. L., Sumiya M., Summerfield J. A., Booy R., Levin M. (1999). Association of variants of the gene for mannose-binding lectin with susceptibility to meningococcal disease. *The Lancet*.

[23] Singh R. S., Walia A. K., Kanwar J. R. (2016). Protozoa lectins and their role in host-pathogen interactions. *Biotechnology Advances*.

[24] Zhou J., Hu M., Li J. (2019). Mannan-binding lectin regulates inflammatory cytokine production, proliferation, and cytotoxicity of human peripheral natural killer cells. *Mediators of Inflammation*.

[25] Wang F., Li Y., Yang C. (2019). Mannan-binding lectin suppresses peptidoglycan-induced TLR2 activation and inflammatory responses. *Mediators of Inflammation*.

[26] Josephs S. F., Ichim T. E., Prince S. M. (2018). Unleashing endogenous TNF-alpha as a cancer immunotherapeutic. *Journal of Translational Medicine*.

[27] Kawasaki Y., Aoki Y., Magata F. (2014). The effect of single nucleotide polymorphisms in the tumor necrosis factor-*α* gene on reproductive performance and immune function in dairy cattle. *Journal of Reproduction and Development*.

[28] Abraham L. J., Kroeger K. M. (1999). Impact of the -308 TNF promoter polymorphism on the transcriptional regulation of the TNF gene: relevance to disease. *Journal of Leukocyte Biology*.

[29] Bouma G., Crusius J. B. A., Oudkerk Pool M. (1996). Secretion of tumour necrosis factor *α* and lymphotoxin *α* in relation to polymorphisms in the TNF genes and HLA-DR alleles. Relevance for inflammatory bowel disease. *Scandinavian Journal of Immunology*.

[30] Olaniyan S. A., Amodu O. K., Bakare A. A., Troye-Blomberg M., Omotade O. O., Rockett K. A. (2016). Tumour necrosis factor alpha promoter polymorphism, TNF-238 is associated with severe clinical outcome of falciparum malaria in Ibadan southwest Nigeria. *Acta Tropica*.

[31] Levesque M. C., Hobbs M. R., O’Loughlin C. W. (2010). Malaria severity and human nitric oxide synthase type 2 (NOS2) promoter haplotypes. *Human Genetics*.

[32] Hetta H. F., Mekky M. A., Khalil N. K. (2015). Association of colonic regulatory T cells with hepatitis C virus pathogenesis and liver pathology. *Journal of Gastroenterology and Hepatology*.

[33] Planche T., Macallan D. C., Sobande T. (2010). Nitric oxide generation in children with malaria and the NOS2G-954C promoter polymorphism. *American Journal of Physiology - Regulatory, Integrative and Comparative Physiology*.

[34] Dzodzomenyo M., Ghansah A., Ensaw N. (2018). Inducible nitric oxide synthase 2 promoter polymorphism and malaria disease severity in children in Southern Ghana. *PLoS One*.

[35] Kun J. F., Mordmüller B., Lell B., Lehman L. G., Luckner D., Kremsner P. G. (1998). Polymorphism in promoter region of inducible nitric oxide synthase gene and protection against malaria. *The Lancet*.

[36] Hobbs M. R., Udhayakumar V., Levesque M. C. (2002). A new NOS2 promoter polymorphism associated with increased nitric oxide production and protection from severe malaria in Tanzanian and Kenyan children. *The Lancet*.

[37] Kwiatkowski D. P. (2005). How malaria has affected the human genome and what human genetics can teach us about malaria. *The American Journal of Human Genetics*.

[38] Abagna H. B., Acquah F. K., Okonu R., Aryee N. A., Theisen M., Amoah L. E. (2018). Assessment of the quality and quantity of naturally induced antibody responses to EBA175RIII-V in Ghanaian children living in two communities with varying malaria transmission patterns. *Malaria Journal*.

[39] Amoah L. E., Abagna H. B., Akyea-Mensah K., Lo A. C., Kusi K. A., Gyan B. A. (2018). Characterization of anti-EBA175RIII-V in asymptomatic adults and children living in communities in the Greater Accra Region of Ghana with varying malaria transmission intensities. *BMC Immunology*.

[40] Badu K., Gyan B., Appawu M. (2015). Serological evidence of vector and parasite exposure in Southern Ghana: the dynamics of malaria transmission intensity. *Parasites & Vectors*.

[41] Afari E. A., Appawu M., Dunyo S., Baffoe-Wilmot A., Nkrumah F. K. (1995). Malaria infection, morbidity and transmission in two ecological zones Southern Ghana. *African Journal of Health Sciences*.

[42] Singh B., Snounou G., Abdullah M. S., Rahman H. A., Bobogare A., Cox-Singh J. (1999). A genus- and species-specific nested polymerase chain reaction malaria detection assay for epidemiologic studies. *The American Journal of Tropical Medicine and Hygiene*.

[43] Amoah L. E., Acquah F. K., Ayanful-Torgby R. (2018). Dynamics of anti-MSP3 and Pfs230 antibody responses and multiplicity of infection in asymptomatic children from southern Ghana. *Parasites & Vectors*.

[44] Amoah L. E., Opong A., Ayanful-Torgby R., Abankwa J., Acquah F. K. (2016). Prevalence of G6PD deficiency and Plasmodium falciparum parasites in asymptomatic school children living in southern Ghana. *Malaria Journal*.

[45] Laishram D. D., Sutton P. L., Nanda N. (2012). The complexities of malaria disease manifestations with a focus on asymptomatic malaria. *Malaria Journal*.

[46] Okell L. C., Bousema T., Griffin J. T., Ouédraogo A. L., Ghani A. C., Drakeley C. J. (2012). Factors determining the occurrence of submicroscopic malaria infections and their relevance for control. *Nature Communications*.

[47] Roman D. N. R., Anne N. N. R., Singh V., Luther K. M. M., Chantal N. E. M., Albert M. S. (2018). Role of genetic factors and ethnicity on the multiplicity of Plasmodium falciparum infection in children with asymptomatic malaria in Yaoundé, Cameroon. *Heliyon*.

[48] Das B. K., Panda A. K. (2015). MBL-2 polymorphisms (codon 54 and Y-221X) and low MBL levels are associated with susceptibility to multi organ dysfunction in P. falciparum malaria in Odisha, India. *Frontiers in Microbiology*.

[49] Wattavidanage J., Carter R., Perera K., Munasingha A., Bandara S., McGuinness D. (1999). TNF*α* 2 marks high risk of severe disease during Plasmodium falciparum malaria and other infections in Sri Lankans. *Clinical and Experimental Immunology*.

[50] Ubalee R., Suzuki F., Kikuchi M. (2001). Strong association of a tumor necrosis factor-*α* promoter allele with cerebral malaria in Myanmar. *Tissue Antigens*.

[51] Abdalla S., Weatherall D. J. (1982). The direct antiglobulin test in P. falciparum malaria. *British Journal of Haematology*.

[52] Grau G. E., Heremans H., Piguet P. F. (1989). Monoclonal antibody against interferon gamma can prevent experimental cerebral malaria and its associated overproduction of tumor necrosis factor. *Proceedings of the National Academy of Sciences*.

[53] Kurtzhals J. A. L., Rodrigues O., Addae M., Commey J. O. O., Nkrumah F. K., Hviid L. (1997). Reversible suppression of bone marrow response to erythropoietin in Plasmodium falciparum malaria. *British Journal of Haematology*.

[54] Goto T., Monk M. (1998). Regulation of X-chromosome inactivation in development in mice and humans. *Microbiology and Molecular Biology Reviews*.

[55] Barakat T. S., Gribnau J. (2012). X chromosome inactivation in the cycle of life. *Development*.

[56] Mpimbaza A., Walakira A., Ndeezi G. (2018). Associations between erythrocyte polymorphisms and risks of uncomplicated and severe malaria in Ugandan children: a case control study. *PLoS One*.

[57] Hwang S., Mruk K., Rahighi S. (2018). Correcting glucose-6-phosphate dehydrogenase deficiency with a small-molecule activator. *Nature Communications*.

[58] Cramer J. P., Mockenhaupt F. P., Ehrhardt S. (2004). iNOS promoter variants and severe malaria in Ghanaian children. *Tropical Medicine and International Health*.

