# Supplementary material for: Association of TNF-Alpha, MBL2, NOS2, and G6PD with Malaria Outcomes in People in Southern Ghana
Source: Genet Res (Camb). 2022 Feb 28;2022:6686406. doi: 10.1155/2022/6686406 (PMC8901335; doi:10.1155/2022/6686406)
Supplement: Supplementary Materials — Additional File S1 Table 1. Primers and restriction enzymes used for P. falciparum species identification and genotyping. Additional File S2 Figure 1. Representative agarose gel images of selected PCR reactions. [file 6686406.f1.zip › 6686406.f1/25.5.2020 Paulina cytokine S1 Table.docx]

**S1 Table: Primers and restriction enzymes used for *P. falciparum* species identification and genotyping**

| Primer name | Primer sequence (5’-3’) | Amplicon size (bp) | Fragment sizes (bp) | Restriction enzyme |
| --- | --- | --- | --- | --- |
| G6PD 2021 | TTACAGCTGTGCCCTGCCCT | 211 | 122, 89 | NIaIII |
| G6PD 2022 | AGGGCAACGGCAAGCCTTAC |  |  |  |
| G6PD 3761 | AGGGCAACGGCAAGCCTTAC | 585 | 402, 187 | FokI |
| G6PD 3762 | CTGCGTTTTCTCCGCCAATC |  |  |  |
| MBL21 | CAGGCAGTTTCCTCTGGAAGG | 340 | 246, 84 | BanI |
| MBL22 | GCACCCAGATTGTAGGACAGAG |  |  |  |
| TNF1 | GGCAATAGGTTTTGAGGGCCATG | 107 | 87, 20 | NcoI |
| TNF2 | CACACTCCCCATCCTCCCTGATC |  |  |  |
| NOS21 | TGTTGGGACGGTGAGATCAAGGT | 1273 | 221, 882, 170 | BsaI |
| NOS22 | CTCATCAAAGGTGGCCGAGAGAT |  |  |  |
| rPLU6 | TTAAAATTGTTGCAGTTAAAACG | 1200 |  |  |
| rPLU5 | CCTGTTGTTGCCTTAAACTTC |  |  |  |
| rFAL1 | TTAAACTGGTTTGGGAAAACCAAATATATT | 200 |  |  |
| rFAL2 | ACACAATGAACTCAATCATGACTACCCGTC |  |  |  |

Bp, base pair
