# Supplementary material for: Association of TNF-Alpha, MBL2, NOS2, and G6PD with Malaria Outcomes in People in Southern Ghana
Source: Genet Res (Camb). 2022 Feb 28;2022:6686406. doi: 10.1155/2022/6686406 (PMC8901335; doi:10.1155/2022/6686406)
Supplement: Supplementary Materials — Additional File S1 Table 1. Primers and restriction enzymes used for P. falciparum species identification and genotyping. Additional File S2 Figure 1. Representative agarose gel images of selected PCR reactions. [file 6686406.f1.zip › 6686406.f1/29.11.21 cytokine gene R2 S2 Fig 1.pdf]

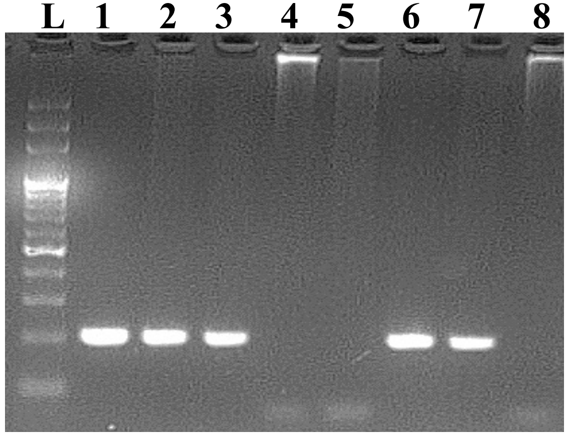

***P. falciparum* 18S rRNA gene**
L: 100 bp ladder,
Lanes 1, 2, 3, 6, 7: positive (200 bp)
Lanes 4, 5, 8: negative

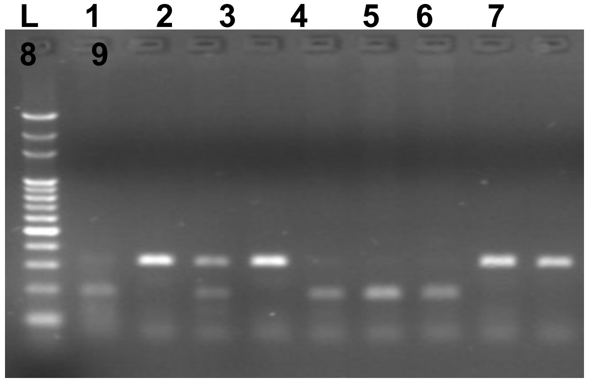

G6PD A376G
L: 100 bp ladder,
Lanes 1,3,5: 376A/G (308, 192, 116 bp)
Lanes 2,4,8,9: 376A/A (308 bp)
Lanes 6,7: 376G/G (192, 116 bp)

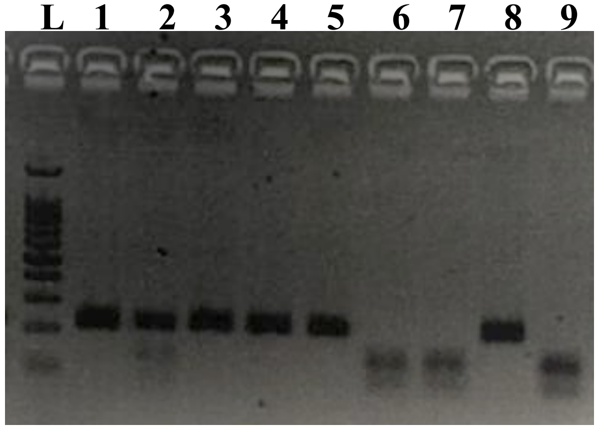

G6PD G202A
L = 100 bp ladder,
Lanes = 1,3,5, 8: 202 G/G (216 bp)
Lanes = 2: 202G/A (216, 81,135 bp)
Lanes = 6,7,9: 202A/A (81, 135 bp)

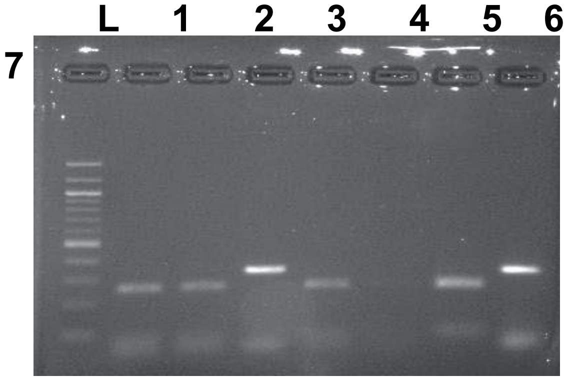

MBL 54-exon 1
L: 100 bp ladder
Lanes 1,2,4, 6: 54G (245 bp, 84 bp)
Lanes 3, 7: 54D (340 bp)

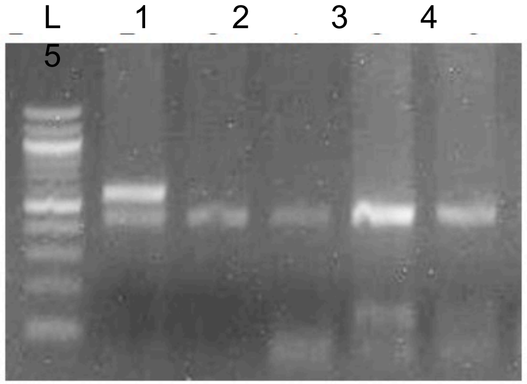

NOS2
L = 100 bp ladder
Lanes 1: 954G/C (573, 437, 136 bp)
Lanes 2-5: 954C/C (437, 136 bp)
